# Alkaloid production of *Solanum elaeagnifolium* Cav from callus for anticancer potential using gene expression of cancer-related genes

**DOI:** 10.1371/journal.pone.0329977

**Published:** 2025-08-22

**Authors:** Abdulkarim Dakah, Iyad Musallam, Raida Wajih Khalil

**Affiliations:** 1 Department of Biotechnology, Faculty of Sciences, University of Kalamoon, Deir attyah, Syria; 2 Biotechnology Research Directorates, National Agricultural Research Center, Amman, Jordan; 3 Department of Biotechnology and Genetic Engineering, Philadelphia University, Amman, Jordan; Canakkale Onsekiz Mart University, TÜRKIYE

## Abstract

Cancer is one of the major diseases that threaten human life and causes death for many people worldwide. Some alkaloids derived from plants show promising potential for cancer treatment. Solanaceae family is of these plants that have promising alkaloid. In this study alkaloid production from *Solanum elaeagnifolium* callus cultures was investigated and their anticancer properties were evaluated. Optimal callus growth and alkaloid production were achieved in media enriched with a combination of BAP and 2,4-D at a ratio of 1.0:1.0 mg/L. LC-MS/MS analysis showed that β-Solamargine, tomatidenol, Solasonine, solanidine and solasodine are the most important alkaloids of *Solanum elaeagnifolium*. Moreover, the analysis revealed that β-Solamargine is the predominant alkaloid (78.7%) in callus extracts. The results of MTT assay, demonstrated that the most effective response were obtained from callus extracts medium containing a balanced concentration of BAP and 2,4-D, and it yielding an IC50 of 6.25 µl/ml. In contrast, lower efficacy was observed with IC50 values of 25 and 50 µl/ml, when callus medium were supplemented with NaCl and yeast extract, respectively. Gene expression analysis shows an increase in the Bax/Bcl-2 ratio following 24 h of the extracts treatment. Along with a down regulation of CDK1 gene expression in comparison to untreated MCF7 cells, the CDK1 levels were elevated. Alkaloids derived from *S. elaeagnifolium* may be a promising candidate for anticancer therapy; further investigation is needed in vivo.

## 1. Introduction

Plant-derived alkaloids have long been recognized for their pharmacological potential, and their ability to modulate cellular pathways offers promising therapeutic paths [1,2]. However, traditional extraction methods from wild plants hadmany challenges including low yields, seasonal variability and sustainability [1,3]. *Solanum elaeagnifolium* Cav., is one of these plants that get a certainattention for its rich list of bioactive steroidal alkaloids and glycol alkaloids such as solamargine and solasonine, which demonstrate cytotoxic effects against multiple cancer cell lines [2,4]. Phytochemical screenings of *S. elaeagnifolium* have identified many pharmacological chemicals including kaempferol derivatives, mangiferin, and glycoalkaloids with documented antiproliferative activity [2,4]. Solamargine and solasonine disrupt mitochondrial membrane potential in cervical (HeLa) and breast cancer cells, inducing apoptosis through caspase activation [2,5]. For industrial purposes, consistent production of bioactive compounds is required. Wild populations plants cannot support the required quantities; therefore a biotechnological intervention was needed [4]. The synergy between recent biotechnological methods and multiomics analyses has significantly enhanced the potential of plant cell cultures in producing alkaloids on a sustainable basis. Modern studies point toward the importance of culture optimization factors, such as airflow and hormone balance, so that maximum production of metabolites can be ensured. As an example, multiomics profiling of tobacco, rice, and bamboo callus cultures revealed species-specific metabolic adjustments upon humidity and airflow alteration with clear differences in Principle component analysis (PCA) extracted meta bolomic clustering following dehydration stress [6]. This highlights the need for tailored environmental regulation in *S. elaeagnifolium* callus systems to stabilize alkaloid biosynthesis. Callus cultures provide a controlled platform for secondary metabolite synthesis, enabling yield optimization through hormonal regulation and elicitation strategies [7]. Plant tissue culture techniques significantly enhanced the sustainable potential of alkaloid production. To success with the production of alkaloid from the lab, optimizing culture conditions is needed. which highlights the need for customized environmental conditions in *S. elaeagnifolium* callus culture to stabilize alkaloid biosynthesis. For many alkaloids, callus cultures provide a good source for secondary metabolite synthesis. For example, *Hyoscyamus aureus* demonstrated that on dry weight bases callus cultures produce more hyoscyamine levels (0.19%) than wild plants (0.17%) [3]. Lupinus demonstrated that root-derived callus cultures treated with 2 mg L ⁻ ¹ 2,4-D and 1 mg L ⁻ ¹ kinetin,produce optimal quinolizidine alkaloid production, with growth kinetics revealing a critical transition to senescence at 18 days [8]. The effect of various auxins (2,4-D, 2,4,5-T, NAA, and IAA) on callus maintenance and initiation of plant cell suspensions, as well as the role of auxins along with cytokinins in growth and productivity of Solasonine by cell suspensions of *Solanum eleagnifolium* Cav., were explored. Calli lines were rendered friable and productive by 2,4-D and 2,4,5-T. Fine and homogeneous cell suspension cultures were attained. Highest growth indices and solasodine productivities of around 0.5 mg 1^−1^ day^−1^ to 0.8 mg 1^−1^ day^−1^ were achieved through 2,4-D (0.5 μM or 5 μM) or NAA (50 μM) [9]. Also Solasodine alkaloids from callus tissues of *Solanum eleagnifolium* Cav. were detected using spectrophotometric and TLC methods. Concentrations ranged from 1.00 to 2.15 mg.g^−1^ DW [10].Such studies providean outline for developing *S. elaeagnifolium* callus protocols. Demonstrating the capabilities of *in vitro* systems to bypass ecological and supply-chain limitations, while enabling genetic and metabolic standardization [1,3]. Also some media in callus induction or in subcultured many times lead to genetic changes in breeding programs [11,12]. Biotechnological approaches like RT-PCR enable precise quantification of alkaloid-induced changes in oncogenic or apoptotic gene expression like p53, Bax/Bcl-2, Recent advances in nano biotechnology further highlight synergistic strategies, such as siRNA co-delivery with alkaloids to suppress HPV oncogenes (E6/E7) in cervical cancer models [5]. These tools not only validate mechanistic pathways but also refine therapeutic efficacy assessments, bridging Phytochemistry and translational oncology [1,5].

This study synthesizes these domains, leveraging callus culture optimization for scalable alkaloid production from S. elaeagnifolium, coupled with RT-PCR-based profiling to elucidate its anticancer mechanisms at the transcriptional level.

## 2. Materials and methods

### 2.1 Plant material and seed preparation

**Fig 1:**
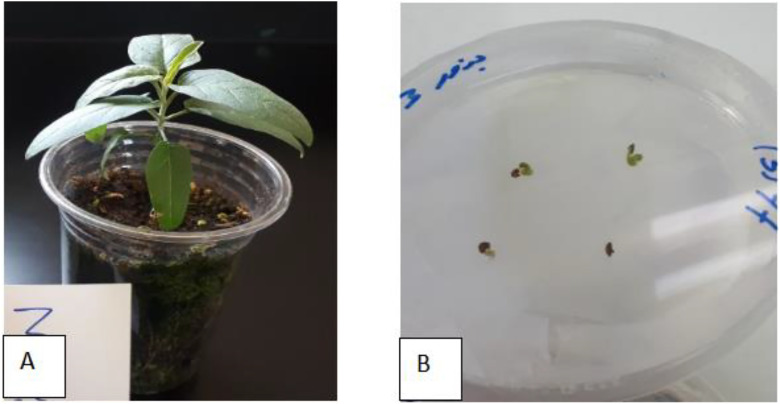
Solanum elaeagnifolium. (A): *Solanum elaeagnifolium* in lab, (B): Germination seeds on ½ MS medium.

*Solanum elaeagnifolium* Cav from the Solanaceae family was selected for this study Fig 1A. The seeds were obtained from the genebank of the National Agricultural Research Center (NARC) in Jordan. Seeds were thoroughly washed with a 0.1% (w/v) Tween and Carbendazim solution for 30 min, treated with 2% sodium hypochlorite solution for 20 min, the surface-sterilized seeds were washed four times with sterile distilled water and placed on 1/2 MS medium [13], Fig 1B. All cultures were incubated at 25 ± 2°C for an 8-h photoperiod with a density of 60 μmol m^−2^ s^−1^, which was provided by LED lamps (24w; Phillips, China).

### 2.2 Callus induction

The calli were induced on MS media containing different concentrations of BAP: 2,4-D (0.5:1.0, 1.0:1.0, 1.0:1.5and 1.0:2.0 mg.L^-1^). The cultures were kept in controlled environment at 25 + 2°C and 16−8 h light/dark regime under LED light. Due to the best calli formed in BAP:2,4-D (1.0:1.0, mg L^-1^) this media were used for callus growth and maintenance. (calli that obtained from roots)

### 2.3 Alkaloids production

MS medium were supplemented with different treatments to investigate their effect on calli growth and production Table 1. The pH of the medium was adjusted to 5.7 before autoclaving. These cultures were regularly sub-cultured at the interval of three weeks on the same medium.

**Table 1:** The different treatments of growth regulators, NaCl and yeast extract supplemented to MS medium to test theireffect on alkaloid production.

Treatment	CGM1	CGM2	CGM3	CGM4
	BAP: 2, 4-D	BAP: 2, 4-D	BAP: 2, 4-D + NaCl	BAP: 2, 4-D + Yeast
**Concentration**	1:1 mg/L	1.5:1.5 mg/L	1.5:1.5 mg/L + 1 g/L	1:1 mg/L + 1.5 g/L

CGM: Callus Growth Media.

### 2.4 Alkaloids extract

Quantification of Alkaloids for each culture was made after 8 weeks of incubation. Three randomized calli tissue samples had been taken and dried in oven at 35°Cfor72 hr, then it was weighted and alkaloid was extracted with Acidic Extraction method according to Miramonte and Flores [14] with some modifications. Briefly: plant material were Mixed with water at a 1:2 ratio (plant-to-water). Sulphur dioxide was added until pH 2–3 is achieved and stir mechanically for 2–6 h. After that the aqueous extract was separate via centrifugation. Calcium hydroxide was added to precipitate alkaloids. The crude precipitate was extract with ethanol, and concentrate to dryness. To compare the quantity of the different alkaloids, seeds of plant *Solanum elaeagnifolium Cav.* were grown in growth room for 60 d (Fig 1). Leaf samples from each species were collected and dried in oven at 35°C for 72 hr, then it was weighted and alkaloid was extracted.

### 2.5 Alkaloids analysis

Liquid -chromatography–mass spectrometry (LC-MS-MS) was used to screen alkaloids in extracts, caffeine was used as internal standard. LC Conditions: Column: C18 reversed-phase (e.g., Gemini NX-C18, 150 mm × 4.6 mm, 3 µm). Mobile Phase: A: 0.2% formic acid in water or 5 mM ammonium carbonate buffer (pH 9). B: Acetonitrile/methanol (1:1 v/v) or pure acetonitrile. Flow Rate: 0.3–0.5 mL/min. MS Parameters, Ionization: ESI + , Detection: MRM mode with optimized transitions (peramine: m/z 249 → 188; ergovaline: m/z 534 → 268). Capillary voltage: 3.5 kV, Source temperature: 300°C, Collision energies: 10–30 eV.

### 2.6 MTT assay

The anticancer activities of the plant extracts were evaluated against one type of cancer cell lines MCF7 by the MTT colorimetric assay according to Dakah and his colleagues [15], stock samples of extracts were serially diluted to be 50, 25, 12.5, 6.25, 3.125, 1.56, 0.78 and 0.39µl/ml. the intensity of the dissolved formazan crystals was quantified using the ELISA plate reader at 540 nm. And the viability was calculated from following formula: Viability% = (Test OD – blank OD)/ (Control OD – blank OD) x 100. Inhibition percentage was calculated as follow: 100 – Viability %.

### 2.7 RT-PCR

Real-Time PCR for five genes (BCL2, BAX, CDK1, P53 and GAPDH as control) were performed. The primers were chosen by specific sequences Table 2. Total RNA was converted to cDNA using The SOLIScript RT cDNA Synthesis Kit according to manufacturers’ instructions. Real-time RT-PCR was performed using SYBR Premix Ex Taq II and ViiA7 real-time PCR system. Data were normalized against GAPDH levels, and the relative fold change was calculated using ViiA7 software. All reactions were performed in triplicate.

**Table 2:** Gene sequences.

Genes	Primers	
	Forward	Reverse
*Bcl-2*	CAGCCTGCAGCTTTGTTTCA	TTTGCCTTTTCCCCTCTCACC
BAX	TGACGGCAACTTCAACTGGG	CACAGCCATCTCTCTCCATGC
CDK1	GAACACCACTTGTCCCTCTAAG	ACATTTTTGGTCCCCGATCC
TP53	GGGATCCAGCATGAGACACTT	GAGTGCTTGGGTTGTGGTGA
GAPDH	GGGAGCCAAAAGGGTCATCA	TCCCATTCCCCAGCTCTCATA

## 3. Results

### 3.1 Callus induction

**Fig 2:**
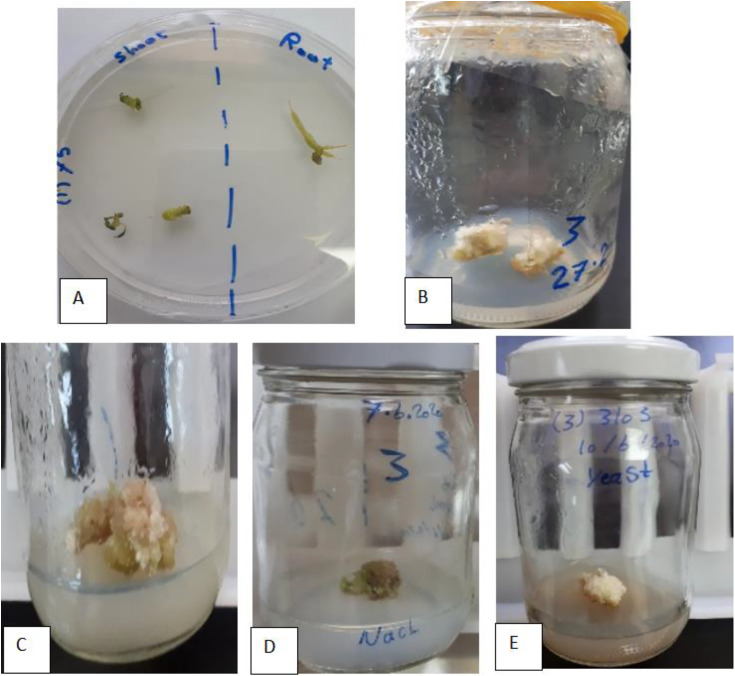
Callus induction. (A) Response of root and shoot of *Solanum elaeagnifolium Cav.* grown on MS medium supplemented with 1.0:1.0, mg L^-1^ of BAP: 2,4-D after2 weeks of Inoculation. (B,C) Response and callus formation of *Solanum elaeagnifolium Cav* on MS medium supplemented with 1.0:1.0, mg L^-1^ of BAP: 2,4-D (CGM1) after eight weeks of Inoculation. (D) Effect of NaCl (CGM3). (E) Effect of yeast extract (CGM4).

Roots were found to response better than shoots for callus induction Fig 2A. The best callus induction occurred in MS medium enriched with BAP and 2,4-D at a ratio of 1.0:1.0 mg L^-1^ Fig 2B and 2C.Due to the best callus formed in previous ratio (BAP:2,4-D at a ratio of1.0:1.0, mg L^-1^),this media was used for callus growth and maintenance. Adding NaCl and yeast extract to the media negatively affects the growth and the diameter of callus (1 cm) compared with other treatments (2 cm) Fig 2D and 2E. (Table 3).

**Table 3:** Effect of the different ratio of growth regulator (BAP:2,4-D), with, and without NaCl and yeast extract supplemented to MS medium on callus diameter, color and softens of Solanum elaeagnifolium Cav.

Treatment Name	M1	M2	M3
Treatment	BAP:2,4-D	BAP: 2,4-D + NaCl	BAP: 2,4-D + Yeast
Concentration	1.5:1.5 mg/L	1.5:1.5 mg/L + 1 g/L	1.0:1.0 mg/L + 1.5 g/L
callus Dimeter (cm)	2	2	1
color and softens	Green-solid	Green-solid	Grey-solid

### 3.2 Alkaloids analysis

**Fig 3:**
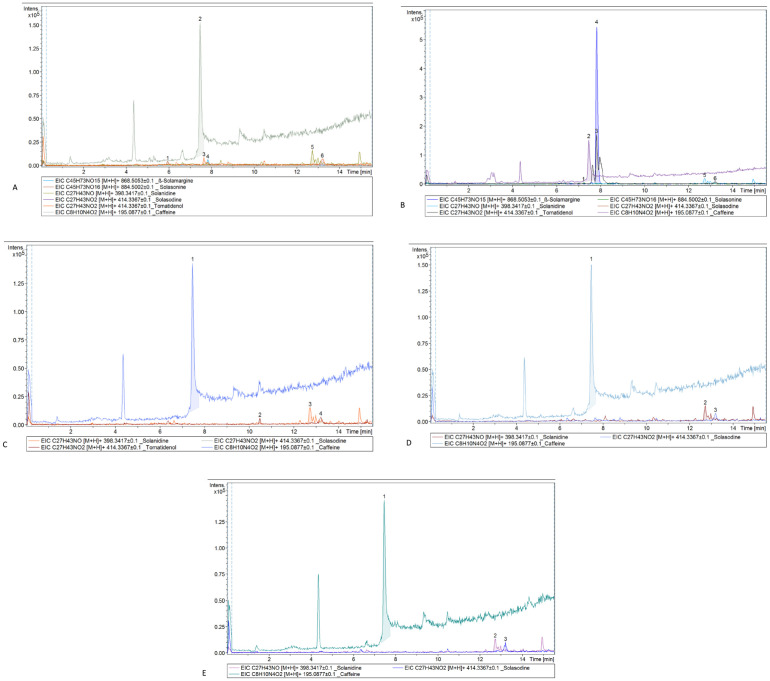
Chromatogram profiles of *Solanum elaeagnifolium.* (A) Extract of leaves. (B) Extract of callus that growth on MS medium supplemented with 1:1 mg/L of BAP: 2, 4-D. (C) Extract of callus that growth on MS medium supplemented with 1.5:1.5 mg/L of BAP:2, 4-D. (D) Extract of callus that growth on MS medium supplemented with 1.5:1.5 mg/L of BAP: 2, 4-D + 1 g/L NaCl. (E) Extract of callus that growth on MS medium supplemented with 1:1 mg/L of BAP: 2, 4-D + 1.5 g/L Yeast.

LC–MS-MS Analysis showed that β-Solamargine, tomatidenol, Solasonine,solanidine and solasodine are the most important alkaloids of *S. elaeagnifolium.*All these compounds were found in the extracts of leaves and callus (grown on M1), but with a different concentration Fig 3A and 3B.The highest concentration (78.7%) of β-Solamargine was found in the callus grown on M1, while the plant leaves gave a concentrations of 13.7%.Solanidine and solasodine were found with different concentration in all extracts of four different treatments Fig 3A–3E.The results showed that increasing concentration of BAP or 2,4-D or adding NaCl or extract of yeast lead to disappearance of β-Solamargine, Solasonine and tomatidenol. (Table 4).

**Table 4:** Alkaloid componentsidentified in *Solanum elaeagnifolium* leaf and callus cultures (CGM1–CGM4) by Liquid -chromatography–mass spectrometry.

Components	Retention time (min)	Leaves	CGM1*	CGM2	CGM3	CGM4
		Area				
Solasonine	5.9	31917	30780	BDL**	BDL	BDL
Caffeine***	7.5	1088486	1088332	1088291	1088454	1088685
tomatidenol	7.8	42658	602771	25739	BDL	BDL
β-Solamargine	7.8	36506	3077263	BDL	BDL	BDL
solanidine	12.7	95043	122987	108632	92914	81425
solasodine	13.2	59386	74263	53589	58371	39973

*CGM1–CGM4: represent variant extract type, ** BDL: Below detection limit, *** Caffeine: Internal standard.

### 3.3 MTT assay

MTT assay showed that the best result was obtained from the extracts of M1 callus with IC_50_ = 6.25 (µl/ml), while a low effect (IC_50_ = 25 and 50 (µl/ml)) was observed from extracts of M2 and M3 callus, respectively. Table 5.

**Table 5:** Anticancer activities of *Solanum elaeagnifolium* alkaloids extracts on death of MCF7 Breast Cancer Cell Line after 24 h of treatment (IC_50_ ± SE).

Cell Line	Extract	*IC_50_ Conc. (µl/ml)
MCF7	Leaves	12.5 ± 0.01^b^
	CGM1**	6.25 ± 0.001^a^
	CGM2	25 ± 0.14^c^
	CGM3	25 ± 0.113^c^
	CGM4	50 ± 0.124^d^

*IC50 values determined using MTT assay. Lower IC50 indicates higher potency.

**CGM1–CGM4 represent specific alkaloid extracted from *Solanum elaeagnifolium*. Units are expressed as µl/ml.

### 3.4 Gene expression (RT-PCR)

**Fig 4:**
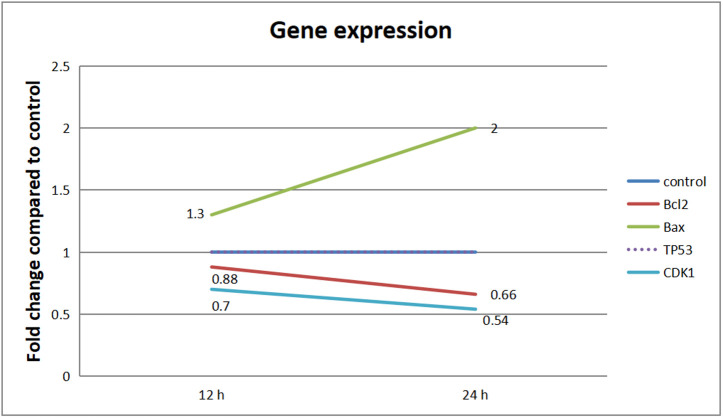
Gene Expression (RT-PCR). The influence of alkaloids extract (CGM1 treatment) on the expression of genes TP53, CDK1, BCL2, and BAX in MCF7 cells.

Extracts of callus that grow on M1 media showed different gene expressions according to the studied gene (Table 6).Untreated MCF7 cells showed a relatively high expression of Bcl-2 which decreased and regulated by 1.5-fold after treatment with extract for 24 hrs. The expression of Bax was increased and up regulated 2 fold after treatment. Increase in the Bax/Bcl-2 ratio after 24 h of extract treatment was noticed, so the extract activates apoptosis. Furthermore, extracts down regulated the expression of *CDK1* gene level compared to untreated MCF7 cells that showed a higher CDK1 expression level. Moreover, theP53gene level wasn’t changed in response to extracts. (Fig 4).

**Table 6:** Gene expression of MCF7 breast cancer cells treated with *solanum elaeagnifolium* callus extracts at 12 and 24 h.

	Time	BCl2	Bax	CDK1	TP53
Untreated MCF7	24 h	↑ High	↓ Low	↑ High	↓ Low
Treated MCF7	24 h	↓ Low	↑ High	↓ Low	↓ Low

Note: Data is presented only for 24 h, as no differences were observed at 12 h.

## 4. Discussion

### 4.1 Callus induction and effect of different treatments

Our study on *Solanum elaeagnifolium* callus induction using BAP:2,4-D ratio of 1.0:1.0 mg/L aligns with trends observed in related *Solanum* species but highlights species-specific variations. The 1:1 BAP:2,4-D ratio diverges from other studies that favor higher auxin concentrations, for instance, *S. virginianum* required 1.0 mg/L BAP + 2.0 mg/L 2,4-D for optimal induction [16], However, this ratio matches trends in *S. lycopersicum*, where balanced ratios improved callus quality [17]. Conversely, this ratio aligns with observations in S. lycopersicum, where balanced auxin ratios have been shown to improve callus quality [17]. Also, *Solanum tuberosum*demonstrated100% callus induction on media with higher auxin ratios, specifically 2, 4-D at 2 mg/L [18]. In another study, *Solanum torvum* achieved 100% callus induction using 1.0 mg/L BA + 0.5 mg/L NAA (stem/leaf explants) [19], suggesting species-dependent auxin: cytokinin ratios. The control treatment (BAP 1.5 mg/L + 2,4-D 1.5 mg/L) and the salt stress treatment (BAP 1.5 mg/L + 2,4-D 1.5 mg/L + 1 g/L NaCl) both produced green, solid calluses with a diameter of 2 cm. This suggests that moderate salt stress at 1 g/L did not adversely affect callus growth or morphology compared to the control. In contrast, the yeast extract treatment (BAP 1.0 mg/L + 2,4-D 1.0 mg/L + 1.5 g/L yeast) resulted in smaller callus (1 cm diameter) with a grey, solid appearance, indicating a possible stress response or altered metabolism. while the callus tissues obtained from Solanum elaeagnifolium in vitro cultures exhibited typical pale yellow to light brown coloration, with generally friable consistency facilitating handling and metabolite extraction. Occasional browning was observed, attributed to oxidative stress and phenolic accumulation, which was minimized by optimizing culture conditions including plant growth regulator concentrations, culture age, and inoculums size [9]. The addition of yeast extract negatively impacted callus growth. However, organic additives such as coconut water often enhances growth [20].This suggests potential species-specific sensitivities to organic supplements or interactions with growth regulators. Callus diameter (1 cm with yeast vs. 2 cm without yeast or with NaCl) highlights potential growth inhibition caused by these additives. The observed reduction in callus diameter aligns with several studies that demonstrate how additives can either enhance or inhibit growth depending on concentration, composition, and interaction with media components. A study on Pluronic F-68 (PF-68) in rice callus found that 0.04% PF-68 improved proliferation by 68%, while 0.10% PF-68 induced oxidative stress and browning [21]. Similarly, yeast extract or related additives may inhibit growth at higher concentrations due to nutrient imbalances or toxicity.In yeast cultivation media, citrate additives (common in yeast extracts) inhibited growth by chelating trace elements (e.g., Fe, Zn), reducing their bioavailability [22]. This mechanism could explain smaller callus diameter sobserved if yeast additives similarly bind essential minerals.

### 4.2 Alkaloids analysis

The LC-MS/MS analysis of Solanum elaeagnifolium alkaloids reveals critical insights into the regulation species-specific biosynthesis regulation and media-dependent variations. Solanidine and solasodine were consistently present across all treatments, in contrast tosolasonine, β-solamargine and tomatidenol, which exhibited sensitivity. Elevated levels of BAP/2,4-D, NaCl, or yeast extract suppressed glycol alkaloids like β-solamargine, suggesting altered flux in the steroidal alkaloid pathway. This observation aligns with previous Solanum biosynthetic studies, where enzymes like GAME12 facilitate nitrogen incorporation into steroidal alkaloids [23] Hormonal imbalances such as excess auxins and cytokinins, likely disrupt this pathway, as observed in *S. nigrum* and *S. tuberosum* [23,24]. Solasodine and solanidine are ubiquitous in Solanum (e.g., *S. erianthum*, *S. torvum*) [25,26], often linked to stress responses and allelopathy [26]. β-solamargine and solasonine are characteristic of *S. melongena* and *S. elaeagnifolium*, though their concentrations vary by tissue type. [2,27]. In contrast to *S. lycopersicum* (tomato), where salinity up regulates glycoalkaloids [23] *S. elaeagnifolium* exhibited suppression under NaCl stress, indicating species-specific metabolic adaptations [26]. Yeast extract enhances solasodine in *S. trilobatum* [25] but inhibits glycoalkaloids in *S. elaeagnifolium*, reflecting divergent regulatory mechanisms [26]. The BAP:2,4-D (1:1) ratio maximizes β-solamargine yield (78.7%), positioning S. elaeagnifolium as a viable source for industrial extraction [26]. Controlled NaCl exposure could fine-tune solanidine/solasodine ratios without suppressing glycoalkaloids [23,26].

### 4.3 MTT assay

The superior activity of our BAP:2,4-D extract suggests that optimized media conditions may enhance glycoalkaloid biosynthesis. In contrast,NaCl or yeast extract supplementation likely induces stress or diluted the synthesis of bioactive compounds synthesis [2,28]. The lower efficacy of NaCl- or yeast extract-supplemented media parallels studies indicating thatabiotic stress alters metabolite profiles. Salt stress in Solanum species often reduces glycoalkaloid yields, which correlates with diminished bioactivity [28]. Our results align with studies showing that plant growth regulators like BAP and 2,4-D enhance secondary metabolite production. Extracts from *Solanum melongena* fruit peel, rich in glycoalkaloids (solamargine, solasonine) exhibited IC_50_ values of 15–20 µg/ml against liver cancer cells, attributed to cell cycle arrest and apoptosis.Solanum glycoalkaloids (e.g., solamargine, solanine) consistently demonstrate dose-dependent cytotoxicity, with Solamargine from *S. melongena* inducing apoptosis in Huh7 cells at low concentrations (IC_50_ 10 µg/ml) [29].

### 4.4 Gene expression (RT-PCR)

The anticancer activity observed in *Solanum elaeagnifolium* callus extracts aligns with findings in other *Solanum* species. Our results indicate that these extracts reduce Bcl-2 expression by 1.5-fold while increasing Bax expression by 2-fold in MCF7 cells, thereby elevating the Bax/Bcl-2 ratio and activating apoptosis [30,31]. This is in aligns with studies on *Solanum aculeastrum*, where steroidal glycosides have been shown induced apoptosis in cancer cells through similar modulation of Bcl-2 family proteins [32]. Our study found no change in p53 expression, suggesting a p53-independent apoptotic pathway. This finding contrasts with research indicating thatBcl-2 over expression inhibits p53-mediated apoptosis in bladder cancer [3,31], highlighting the species- and context-dependent mechanisms involved. The observed down regulation of CDK1 parallels studies indicating thatBcl-2 modulation can indirectly affects cell cycle progression. For example, Bcl-2 inhibition in colon cancer has been shown to reduced proliferation independently of p53 [33], which mirrors our extract’s ability to suppress CDK1 without involving p53. In contrast, the stress-induced terpene biosynthesis in *S. elaeagnifolium*’s [30] may account forits bioactivity, as terpenes like (E)-caryophyllene are associated with stress responses that could synergize with apoptotic signaling. Compared to flavonoid rich Solanum species that inhibit Bcl-2 via p53 [34], our results suggest that distinct phytochemical drivers, such as terpenes or alkaloids, are present in *S. elaeagnifolium* extracts. This mechanistic divergence emohasizesthe chemical diversity within the genus while reinforcing its broad anticancer potential through shared apoptotic targets like Bax/Bcl-2 [31,32]. Further comparative studies should investigate whether these effects correlate with specific metabolites identified in the transcriptomes of *S. elaeagnifolium* [4,30].

## 5. Conclusion

This study successfully employed biotechnological tools to produce and analyze alkaloids from Solanum elaeagnifolium callus cultures, demonstrating their potential anticancer activity. The use of BAP:2,4-D at a 1:1 ratio optimized callus growth and alkaloid production, with β-Solamargine being the most prevalent. Increasing concentration of plant growth regulator or adding NaCl and yeast extract have negatively effects on growth of calli and contents of alkaloids. MTT assay and gene expression analysis using RT-PCR highlighted the cytotoxic and anticancer effects of these alkaloids against MCF7 cells. The findings suggest that alkaloids from S. elaeagnifolium, especially β-Solamargine, tomatidenol, Solasonine and solanidine and solasodine, could be valuable anticancer agents. Further in vivo studies are necessary to confirm their efficacy and safety for clinical applications.

## References

[1] Koirala M, Karimzadegan V, Liyanage NS, Mérindol N, Desgagné-Penix I. Biotechnological approaches to optimize the production of Amaryllidaceae alkaloids. Biomolecules. 2022;12(7):893. doi: 10.3390/biom12070893 35883449 PMC9313318

[2] Hernández O L. Bioguided fractionation from Solanum elaeagnifolium to evaluate toxicity on cellular lines and breast tumor explants. Vitae. 2017;24(2):124–31. doi: 10.17533/udea.vitae.v24n2a05

[3] Besher S, Al-Ammouri Y, Murshed R. Production of tropan alkaloids in the in vitro and callus cultures of Hyoscyamus aureus and their genetic stability assessment using ISSR markers. Physiol Mol Biol Plants. 2014;20:343–9. 25049461 10.1007/s12298-014-0242-6 PMC4101135

[4] Kobisi ANA, Balah MA, Hassan AR. Bioactivity of silverleaf nightshade (Solanum elaeagnifolium Cav.) berries parts against Galleria mellonella and Erwinia carotovora and LC-MS chemical profile of its potential extract. Sci Rep. 2024;14(1):18747. 39138246 10.1038/s41598-024-68961-z PMC11322330

[5] Xie W, Xu Z. (Nano)biotechnological approaches in the treatment of cervical cancer: integration of engineering and biology. Front Immunol. 2024;15:1461894. doi: 10.3389/fimmu.2024.1461894 39346915 PMC11427397

[6] Kim J-S, Sato M, Kojima M, Asrori MI, Uehara-Yamaguchi Y, Takebayashi Y, et al. Multiomics-based assessment of the impact of airflow on diverse plant callus cultures. Sci Data. 2025;12(1):197. doi: 10.1038/s41597-025-04518-7 39900939 PMC11790825

[7] Fazili MA, Bashir I, Ahmad M, Yaqoob U, Geelani SN. In vitro strategies for the enhancement of secondary metabolite production in plants: a review. Bull Natl Res Cent. 2022;46(1):35. doi: 10.1186/s42269-022-00717-z 35221660 PMC8857880

[8] Salcedo-Morales G, et al. Germination and callus induction for alkaloid production of two Lupinus species under in vitro conditions. Polibotánica. 2024;57:183–97.

[9] Alvarez MA, Nigra HM, Giulietti AM. Solasodine production by Solanum eleagnifolium Cav. in vitro cultures: Influence of plant growth regulators, age and inoculum size. Large-scale production. Nat Prod Lett. 1993;3(1):9–19.

[10] Nigra H, Caso O, Giulietti A. Production of solasodine by calli from different parts of Solanum eleganifolium Cav. plants. Plant Cell Rep. 1987;6:135–7. 24248495 10.1007/BF00276671

[11] Laila LA, Zaid SH, Al-Biski F, Dakah A. Regeneration of selected callus of three potato cultivars (Solanum tuberosum.L) and studying their tolerance to drought stress. BMC Plant Biol. 2025;25(1):460. doi: 10.1186/s12870-025-06512-y 40211133 PMC11983820

[12] Dakah A, Suleiman M, Zaid S. Genetic relationship among wild medicinal genotypes of Ziziphora canescens Benth. and Ziziphora tenuior L. and detection of genetic variations resulted from tissue culture, salinity and pH media. Am J Agric Biol Sci. 2015;10(3):144–56.

[13] Murashige T, Skoog F. A revised medium for rapid growth and bio assays with tobacco tissue cultures. Physiol Plant. 1962;15(3):473–97. doi: 10.1111/j.1399-3054.1962.tb08052.x

[14] Miramonte LE, Flores HJ. Process for isolation of solanum alkaloids from solanum plants. Google Patents; 1968.

[15] Dakah A, et al. Effect of Olea europaea L. and Juglans regia L. extracts on human cancer cell line viability with studying of hypoglycemic and antiglycation properties. J Pharm Res Int. 2021:1–7.

[16] Shete R, Jadhav A, Pandhure N. In vitro callus induction in Solanum virginianum L. Int J Sci Res. 2015;5(7).

[17] Kumari A, Bhatti SS, Nagpal AK, Katnoria JK. Antioxidative response appraisal of callus cultures of Solanum lycopersicum L. under heavy metal stress. Nucleus. 2024;68(1):1–11. doi: 10.1007/s13237-023-00463-1

[18] Qureshi MA, Ahmed R, Ahmed B, Khan TU, Yasin M. Effect of growth regulators on callus induction and plant regeneration in potato (Solanum tuberosum L.) explants. SJA. 2023;39(1):140–46. doi: 10.17582/journal.sja/2023/39.1.140.146

[19] Qin Y-L, Shu X-C, Zhuang W-B, Peng F, Wang Z. High efficiency callus induction and regeneration of solanum torvum plants. HortScience. 2017;52(12):1755–8. doi: 10.21273/hortsci12232-17

[20] Nambiar N, Tee C, Maziah M. Effects of organic additives and different carbohydrate sources on proliferation of protocormlike bodies in ‘Dendrobium’ Alya Pink. Plant Omics. 2012;5(1):10–8.

[21] Kok AD-X, Mohd Yusoff NF, Sekeli R, Wee C-Y, Lamasudin DU, Ong-Abdullah J, et al. Pluronic F-68 improves callus proliferation of recalcitrant rice cultivar via enhanced carbon and nitrogen metabolism and nutrients uptake. Front Plant Sci. 2021;12:667434. doi: 10.3389/fpls.2021.667434 34149763 PMC8207202

[22] Hahn-Hägerdal B, Karhumaa K, Larsson CU, Gorwa-Grauslund M, Görgens J, van Zyl WH. Role of cultivation media in the development of yeast strains for large scale industrial use. Microb Cell Fact. 2005;4:31. doi: 10.1186/1475-2859-4-31 16283927 PMC1316877

[23] Grzech D, Smit SJ, Alam RM, Boccia M, Nakamura Y, Hong B, et al. Incorporation of nitrogen in antinutritional Solanum alkaloid biosynthesis. Nat Chem Biol. 2025;21(1):131–42. doi: 10.1038/s41589-024-01735-w 39271954 PMC11666457

[24] Elizalde-Romero CA, Montoya-Inzunza LA, Contreras-Angulo LA, Heredia JB, Gutiérrez-Grijalva EP. Solanum fruits: phytochemicals, bioaccessibility and bioavailability, and their relationship with their health-promoting effects. Front Nutr. 2021;8:790582. doi: 10.3389/fnut.2021.790582 34938764 PMC8687741

[25] Senizza B, Rocchetti G, Sinan KI, Zengin G, Mahomoodally MF, Glamocilja J, et al. The phenolic and alkaloid profiles of Solanum erianthum and Solanum torvum modulated their biological properties. Food Biosci. 2021;41:100974. doi: 10.1016/j.fbio.2021.100974

[26] Antodiadis C, Vasilakoglou I, Dimos E, Adamou V, Dhima K. Potential subterranean interference of Solanum elaeagnifolium, commonly known as silverleaf nightshade, on durum wheat. Span J Agric Res. 2024;22(2):e1002. doi: 10.5424/sjar/2024222-20723

[27] Manoharan R, Nair CS, Eissa N, Cheng H, Ge P, Ren M, et al. Therapeutic potential of Solanum alkaloids with special emphasis on cancer: a comprehensive review. Drug Des Devel Ther. 2024;18:3063–74. doi: 10.2147/DDDT.S470925 39050799 PMC11268566

[28] Winkiel MJ, Chowański S, Słocińska M. Anticancer activity of glycoalkaloids from Solanum plants: a review. Front Pharmacol. 2022;13:979451. doi: 10.3389/fphar.2022.979451 36569285 PMC9767987

[29] Fekry MI, Ezzat SM, Salama MM, Alshehri OY, Al-Abd AM. Bioactive glycoalkaloides isolated from Solanum melongena fruit peels with potential anticancer properties against hepatocellular carcinoma cells. Sci Rep. 2019;9(1):1746. doi: 10.1038/s41598-018-36089-6 30741973 PMC6370831

[30] Tsaballa A, Nikolaidis A, Trikka F, Ignea C, Kampranis SC, Makris AM, et al. Use of the de novo transcriptome analysis of silver-leaf nightshade (Solanum elaeagnifolium) to identify gene expression changes associated with wounding and terpene biosynthesis. BMC Genomics. 2015;16(1):504. doi: 10.1186/s12864-015-1738-3 26149407 PMC4492009

[31] Miyake H, Hanada N, Nakamura H, Kagawa S, Fujiwara T, Hara I, et al. Overexpression of Bcl-2 in bladder cancer cells inhibits apoptosis induced by cisplatin and adenoviral-mediated p53 gene transfer. Oncogene. 1998;16(7):933–43. doi: 10.1038/sj.onc.1201602 9484785

[32] Kowalczyk T, Merecz-Sadowska A, Rijo P, Mori M, Hatziantoniou S, Górski K, et al. Hidden in plants-A review of the anticancer potential of the Solanaceae family in in vitro and in vivo studies. Cancers (Basel). 2022;14(6):1455. doi: 10.3390/cancers14061455 35326606 PMC8946528

[33] Zinkel S, Gross A, Yang E. BCL2 family in DNA damage and cell cycle control. Cell Death Differ. 2006;13(8):1351–9. doi: 10.1038/sj.cdd.4401987 16763616

[34] Rahman N, Khan H, Zia A, Khan A, Fakhri S, Aschner M, et al. Bcl-2 modulation in p53 signaling pathway by flavonoids: a potential strategy towards the treatment of cancer. Int J Mol Sci. 2021;22(21):11315. doi: 10.3390/ijms222111315 34768743 PMC8582810

